# Effect of Distillation Time on the Yield and Chemical Composition of Leaf Essential Oil from *Thuja occidentalis* L.

**DOI:** 10.3390/plants15121885

**Published:** 2026-06-17

**Authors:** Chanjoo Park, Nahyun Kim, Soo-Kyeong Jang, Mi-Jin Park

**Affiliations:** Forest Industrial Materials Division, Forest Products and Industry Department, National Institute of Forest Science, Seoul 02455, Republic of Korea; chanjoopark515@korea.kr (C.P.); knh1125@korea.kr (N.K.); skjang05@korea.kr (S.-K.J.)

**Keywords:** distillation time, essential oils, *Thuja occidentalis* L., yield, thujone

## Abstract

Distillation is widely used for essential oils extraction, and distillation time (DT) influences the quality and quantity of oils. This study presents the effects of 14 DTs (1, 3, 5, 10, 20, 40, 80, 120, 160, 200, 240, 280, 360, and 480 min) on leaf oils from *Thuja occidentalis* L., aiming to maximise the yield and modulate chemical profiles. Also, a safety assessment of *T. occidentalis* oils obtained at different DTs was conducted based on thujone levels under International Fragrance Association (IFRA) guidelines. Oil yield varied with DT, reaching a maximum at 40–80 min (0.54 ± 0.04% and 0.59 ± 0.02%, respectively). Monoterpenes (sabinene, fenchone, and thujone) decreased with prolonged DT, whereas higher molecular weight compounds, including sesquiterpenes (caryophyllene) and diterpenes (hibaene and rimuene) increased. Principal component analysis (PCA) grouped samples into three stages, early (1–10 min), mid (20–80 min), and late (120–480 min), with clear compositional separation (PC1: 68.4%, PC2: 11.8%). As DT increased, the fragrance profile progressively shifted from monoterpene-rich characteristics in the early-stage oils to more persistent aromas associated with sesquiterpenes (caryophyllene, α-cadinol, and α-eudesmol) and diterpenes (hibaene and rimuene) in the late-stage oils. Safety assessment revealed that early-stage oils contained high thujone levels (67.19–71.88%), limiting their allowable use. In contrast, prolonged DTs (≥200 min) reduced thujone content, increasing permissible usage across multiple IFRA categories. Overall, DT significantly influences the oil yield, chemical composition, and regulatory applicability of *T. occidentalis* oils, with extended distillation enhancing formulation flexibility in cosmetic and fragrance applications.

## 1. Introduction

*Thuja occidentalis* L. (Cupressaceae) is a plant native to North America, and is widely cultivated as an ornamental plant in many parts of the world [1,2,3]. It has coniferous pyramidal features, with flattened branches and twigs in one plane, bearing small scale-like leaves [4]. Historically, *T. occidentalis* oils have been used in folk medicine. Owing to the bioactive component of thujone, these oils have been applied for the treatment of hepatoprotection, bronchial catarrh, rheumatism, psoriasis, and even uterine carcinomas [1]. In South Korea, *T. occidentalis* is commonly planted as a hedge and ornamental tree due to its radially spreading branches, pleasant fragrance, and dense foliage [5]. *T. occidentalis* oils may be a promising candidate for commercial development owing to the stable availability of plant materials from established plantations throughout South Korea. Furthermore, unused forest biomass generated during pruning activities could be utilised as a sustainable feedstock for essential oil production, enhancing resource efficiency.

The essential oils of *T. occidentalis* were extracted from heartwood and leaf parts. Andersen et al. [6] studied the wood oil of *T. occidentalis*, suggesting the utilisation of the waste materials from the cedar shingle industry. In wood-derived oils, occidentalol (20–50%) was identified as the major component, with its concentration varying depending on DT. In contrast, leaf oils are predominantly composed of oxygenated monoterpenes (64.8–87.3%), among which α-thujone (20.1–61.0%) is the most abundant, followed by β-thujone (3.6–10.7%), sabinene (3.0–9.3%), and fenchone (4.9–7.7%) [1,3,7,8]. Furthermore, leaf oils extracted from *T. occidentalis* exhibit various biological activities, including insecticidal activity against mosquitoes and broad antimicrobial activity [5]. Notably, thujone, a major monoterpene constituent, is used pharmacologically as an active ingredient in nasal decongestants and cough suppressants, and is also widely applied in perfumes, shoe polishes, and soaps [9]. However, its use is restricted under International Fragrance Association (IFRA) standards. Specifically, the maximum acceptable thujone concentration in the finished product (%) is limited due to safety concerns [10]. Although some essential oils have been granted GRAS (Generally Regarded as Safe) status by Food and Drug Administration of US [11,12]. Therefore, evaluating the regulatory applicability of *T. occidentalis* oils is necessary to ensure their safe use.

The yield and chemical composition of essential oils are influenced by multiple factors, including genetic variation, plant nutrition, fertiliser application, geographic location, and drying and storage conditions [13,14,15,16]. Among these, DT is a critical parameter that directly affects both oil yield and compositional profile. However, to date, no studies have investigated the effect of DT on *T. occidentalis* oils. In contrast, several studies on commercially important essential oils, such as peppermint, lemongrass, and palmarosa, have demonstrated significant DT-dependent variations [17]. PCA were used as a tool, to demonstrate variation in the composition of different times of essential oils [18]. In particular, PCA enables the visualisation of relationships among major compounds in a time-dependent manner and facilitates the interpretation of associated fragrance characteristics based on the dominant component in oils. Understanding the influence of DT on essential oil yield and composition can provide valuable insights for optimising industrial production. Such findings may enable producers to enhance oil yield, tailor chemical composition, and reduce energy consumption during the distillation process [17].

Therefore, this study aimed to evaluate the effects of DT on *T. occidentalis* oils in terms of yield, chemical composition, and regulatory applicability. Ultimately, manipulation of DT enables effective control of oil yield and quality, thereby enhancing the feasibility of commercial production of *T. occidentalis* oils.

## 2. Results

### 2.1. The Oil Yield and Chemical Profile of T. occidentalis Oils Extracted by Hydrodistillation

**Figure 1:**
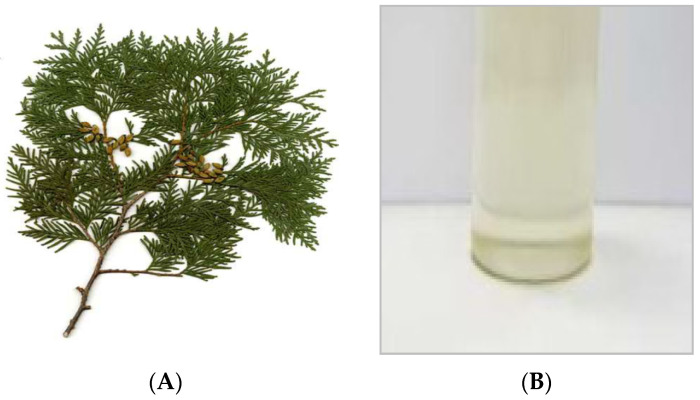
Fresh leaves (**A**) and the colour of leaf oils (**B**) of *T. occidentalis*.

*T. occidentalis* essential oils were extracted until no further oil could be obtained. No additional oils were obtained after 26 h of DT; therefore, the process was terminated at that time. The oil yield of *T. occidentalis* was 2.20%. The *T. occidentalis oils* was extracted from leaves (Figure 1A) and it appeared colourless to pale yellow to the unaided eye (Figure 1B).

As shown in Table 1, the chemical profile of *T. occidentalis* oils revealed 49 components, accounting for 96.84%% of the total oils. The *T. occidentalis* oil was predominantly composed of monoterpenes and oxygenated monoterpenes, with α-thujone (45.52%) identified as the major constituent, followed by fenchone (10.17%) and β-thujone (6.23%). Other notable monoterpene hydrocarbons included sabinene (2.83%), camphene (2.26%), β-phellandrene (1.83%), α-pinene (1.71%), and γ-terpinene (1.38%). Among oxygenated monoterpenes, camphor (2.40%), terpinene-4-ol (2.42%), α-terpineol (0.45%), and borneol derivatives (endo-borneol, 0.17%) were also present. In addition to monoterpenes, sesquiterpenes were detected in lower amounts, including caryophyllene (2.27%), caryophyllene oxide (1.05%), humulene (0.17%), and δ-cadinene (0.09%), along with oxygenated sesquiterpenes such as acorenol (0.28%), α-cadinol (0.15%), and α-eudesmol (0.16%). Furthermore, diterpenes were identified, including hibaene (2.57%), rimuene (1.38%), and kaur-16-ene (0.56%). Overall, the chemical profile of *T. occidentalis* oil was characterised by a strong dominance of thujone-type monoterpenes, particularly α-thujone (45.52%).

### 2.2. Effects of the Length of the DT on the Oil Yield and Selected Components of T. occidentalis Oils

**Figure 2:**
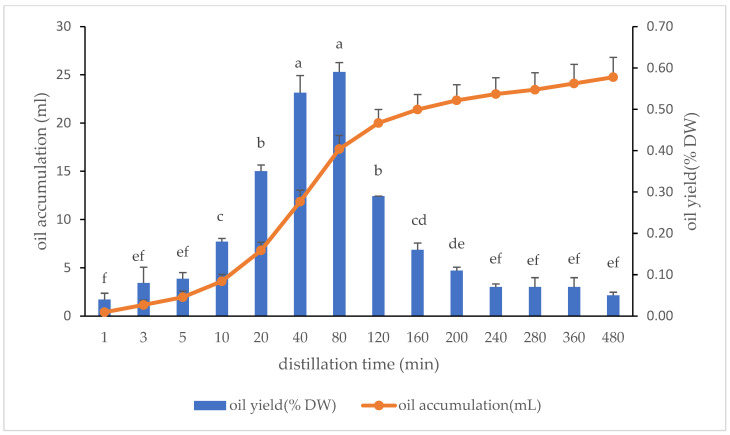
Fractional oil yield (%) and cumulative oil volume (mL) of *T. occidentalis* oils during 8 h of hydrodistillation. Values were expressed as means ± SD (*n* = 3). Values with different letters were significantly different at *p* < 0.05 as analysed by Tukey’s multiple range test of specialised fractional distillation for achieving the targeted yield. Fractional yield represents the percentage of oil obtained in each distillation interval relative to dry weight, while cumulative volume indicates the total collected oil over time.

The effect of DT on *T. occidentalis* essential oil yield and cumulative oil is presented in Figure 2. The oil yield exhibited a strong dependence on DT, with a rapid increase during the early and mid-distillation phases. The highest oil yield was observed at 40 and 80 min (0.54 ± 0.04% and 0.59 ± 0.02%, respectively) after which a gradual decline was noted despite continued distillation. Specifically, the oil yield declined progressively with extended distillation, dropping to less than 10% beyond 160 min (0.16 ± 0.02%) and reaching minimal levels at 480 min (0.05 ± 0.01%). In contrast, cumulative oil accumulation increased continuously throughout the distillation process. The oil volume rose sharply up to 80 min, followed by a gradual increase until reaching a plateau at approximately 360–480 min (around 24–25 mL).

As shown in Table 2, the selected bioactive components (sabinene, fenchone, α-thujone, caryophyllene, hibaene, and rimuene) in *T. occidentalis* oils collected at different DT were identified. Although the selected compounds represent a portion of the total essential oil composition, they were intentionally chosen as representative markers to elucidate compositional dynamics during the distillation process. Rather than reflecting the overall chemical profile of *T. occidentalis* oils, these components provide insight into time-dependent variation patterns. Specifically, monoterpenes (e.g., sabinene, fenchone, and thujone isomers) exhibited a decreasing trend with prolonged DT, whereas higher molecular weight compounds such as sesquiterpenes (caryophyllene) and diterpenes (hibaene and rimuene), increased progressively. Therefore, the selected components were used as indicative compounds to demonstrate how DT influences the sequential release of volatile versus less-volatile constituents, rather than to represent the quantitative composition of the whole essential oil of *T. occidentalis*.

The monoterpenes were predominant during the early stages of distillation (1–40 min). In particular, α-thujone was the major component, ranging from 60.30 ± 1.87% to 64.30 ± 1.32% during the initial phase (1–5 min). However, its content decreased progressively with increasing DT, dropping sharply after 120 min and reaching 0.90 ± 0.04% at 480 min. A similar declining trend was observed for β-thujone, which decreased from 6.89 to 7.60% in the early fractions to 0.18 ± 0.02% at 480 min. Other monoterpenes, including sabinene and fenchone, also exhibited gradual reductions over time, indicating that these volatile compounds were primarily recovered during the early distillation stage. In contrast, sesquiterpene content showed a relatively stable pattern during the early and mid-stages, followed by a gradual increase at longer DT. Caryophyllene remained at 1.90–2.14% up to 200 min, then increased significantly, reaching 3.12 ± 0.10% at 480 min. Diterpenes displayed a markedly different trend compared to monoterpenes. Both hibaene and rimuene were present at low levels in the early stages (0.27 ± 0.06% and 0.17 ± 0.04% at 1 min, respectively) but increased continuously with the prolonged DT. For example, hibaene content rose sharply after 160 min, reaching 6.39 ± 0.78% at 480 min.

### 2.3. Multivariate Analysis of DT-Dependent Chemical Component of T. occidentalis Oils

**Figure 3:**
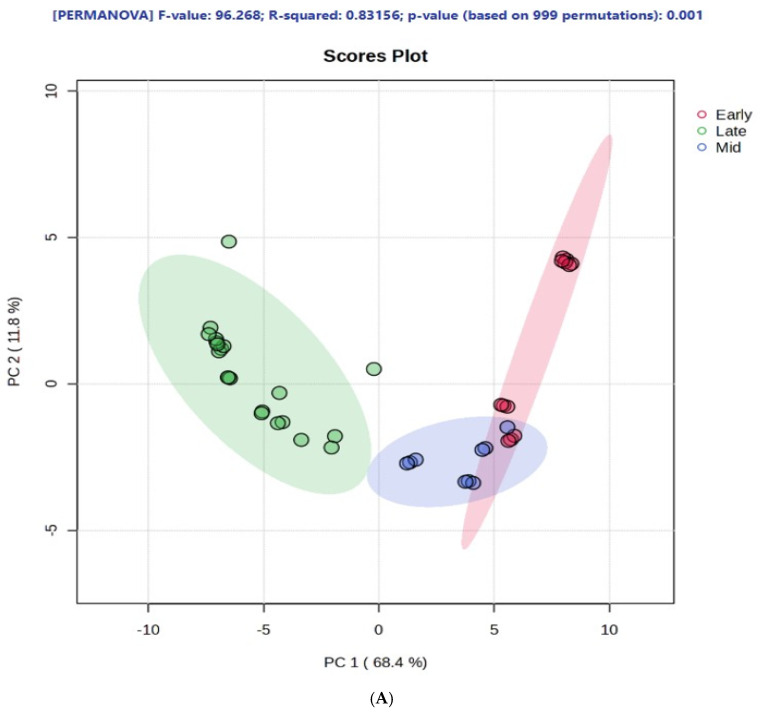

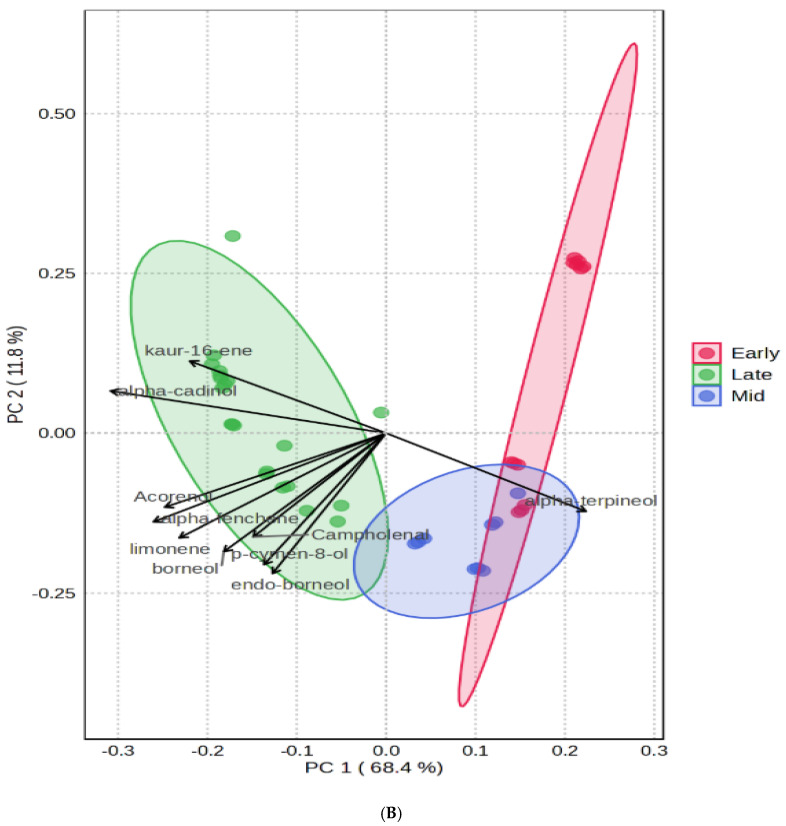
Principal component analysis (PCA) of *T. occidentalis* oil samples. (**A**) The score plot of PCA. (**B**) The biplot of PCA. Data were log-transformed and auto-scaled before clustering. Euclidean distance and Ward’s linkage method were used. Heatmap of the top 25 most discriminative components showing relative abundance patterns across the three stages. The data represent group-averaged values for each stage (early, mid, and late). Specifically, samples were grouped into three temporal categories according to DT: early stage (1, 3, 5, and 10 min), mid stage (20, 40, and 80 min), and late stage (120, 160, 200, 240, 280, 360, and 480 min).

**Figure 4:**
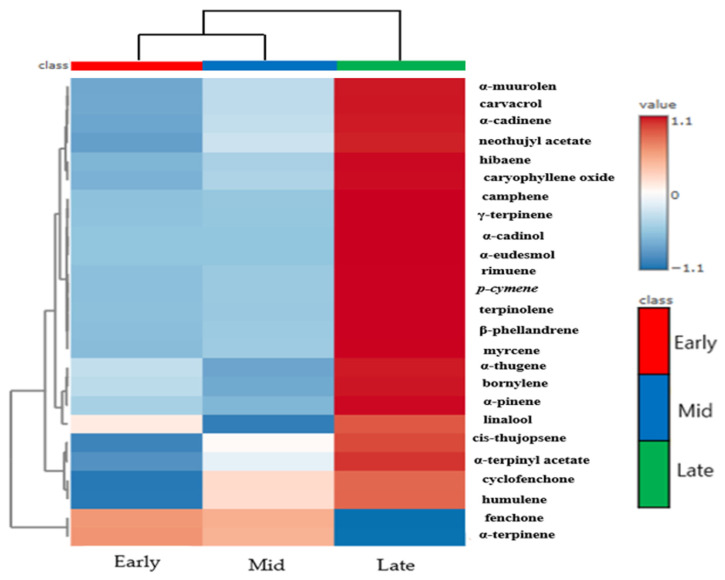
Hierarchical clustering heatmap of major volatile compounds in *T. occidentalis* essential oils obtained at different DTs (Early vs. Mid vs. Late).

From a sensory perspective, to further distinguish fragrance differences among essential oils obtained from different distillations, PCA was performed based on their chemical profiles. To facilitate interpretation of multivariate patterns and reduce visual complexity, the 14 different DT points were grouped into three stages based on DT: early stage (1, 3, 5, and 10 min), mid stage (20, 40, and 80 min), and late stage (120, 160, 200, 240, 280, 360, and 480 min). This grouping was further supported by PCA (Figure 3) and HCA results (Figure 4), which showed clear separation of samples into three distinct compositional clusters.

As shown in Figure 3, the accumulated contribution rate of the first two principal components (PC 1: 68.4%, PC2: 11.8%) was 80.2% of the total variance, indicating that the PCA model sufficiently represented the variability of the samples and allowed clear discrimination. Specifically, the distinct separation of the early, mid, and late distillation stages was statistically validated by Permutational multivariate analysis of variance (F = 96.268, R^2^ = 0.83156, *p* = 0.001), indicating significant differences in the chemical composition of the oils. Late-stage (120, 160, 200, 240, 280, 360, and 480 min) samples were distinctly separated from early (1, 3, 5, and 10 min) and mid stages (20, 40, and 80 min) along PC1, while early and mid-samples were further discriminated along PC2, supporting the classification of samples into three temporal groups. This classification was further supported by HCA, which revealed a consistent grouping pattern, with early-, mid-, and late-stage samples forming distinct clusters based on their chemical profiles (Figure 4).

As shown in Table 3, PC1 was mainly associated with α-terpinene, α-terpineol, fenchone, α-thujone, β-thujone, and camphor, whereas PC2 was largely influenced by endo-borneol, α-fenchene, 3-carene, α-terpineol, and camphor.

The PCA bioplot demonstrated a progressive compositional shift during distillation. Early stage samples were positioned on the positive side of PC1 and were strongly associated with oxygenated monoterpenes, particularly α-terpineol. In contrast, late-stage samples were located on the negative side of PC1 and were correlated with monoterpene hydrocarbons, including limonene and α-fenchene, as well as higher molecular weight compounds such as kaur-16-ene. Mid-stage samples occupied an intermediate position, indicating a transitional chemical profile during the distillation process.

HCA further supported the PCA results by clearly separating the oils into early, mid, and late distillation stages (Figure 4). The early-stage cluster was primarily associated with monoterpene-rich constituents, including α-terpinene, fenchone, cyclofenchone, α-terpinyl acetate, and linalool. In contrast, the late-stage cluster was characterised by higher levels of sesquiterpenes and diterpenes, such as α-muurolene, carvacrol, α-cadinene, α-cadinol, α-eudesmol, and rimuene.

### 2.4. Safety Assessment of T. occidentalis Oils in Relation to Thujone Levels and Regulatory Limits for the Finished Product

The maximum allowable use levels of *T. occidentalis* oils in finished products were estimated across IFRA Categories 1–12 based on the thujone content of oils obtained at DTs (Table 4). At DTs of 1–40 min, the essential oils exhibited extremely high thujone contents (67.19–71.88%), resulting in highly restricted maximum allowable use levels in low-threshold IFRA categories (Table 4). For example, in Category 1 (limit: 0.11%), the maximum allowable use level was limited to approximately 0.15–0.16%, indicating a very narrow formulation range. Similarly, in other restrictive categories such as Category 5(A), 5(C), and 11(A), the allowable levels were below 0.01%, effectively limiting their practical application. With prolonged distillation, the thujone content decreased substantially, leading to increased allowable use levels. At intermediate DTs (80–160 min), moderate increases in formulation flexibility were observed. For example, at 160 min, the allowable use level in Category 1 increased to 0.33%, representing more than a two-fold increase compared with early-stage oils. At prolonged DTs (≥200 min), the reduction in thujone content resulted in significantly enhanced applicability across multiple IFRA categories. Notably, at 200 min, the allowable use level in Category 1 reached 0.63%, while further increases were observed at 240 min (1.38%) and 480 min (10.19%). In less restrictive categories, several values exceeded 100%, indicating no practical limitation on essential oil usage.

## 3. Discussion

### 3.1. Evaluation of Oil Yield and Chemical Composition of T. occidentalis Oils

The essential oil of *T. occidentalis* used as the control in this study was extracted until no further oil could be obtained (26 h distillation). The control oil yield was 2.82%. The fragrance characteristic of *T. occidentalis* oils is camphoraceous, with a sharp and cooling aroma reminiscent of peppermint, accompanied by green and herbal notes. Especially, woody and powdery nuances become more prominent, while the camphor-like sharpness gradually diminishes [19].

Drying is considered the most common and fundamental technique for the post-harvest preservation of herbs and is regarded as a good process to retain bioactive compounds [20]. However, the quantity and quality of essential oils may change during drying, as certain compounds may increase or decrease and new compounds may be formed [21,22]. Likewise, *T. occidentalis* leaf oils showed the variation depending on the drying conditions of the plant materials before distillation. Fresh *T. occidentalis* contains approximately 0.6% essential oil (on a dry matter basis), and the oil from fresh leaves is composed primarily of monoterpenes, including thujone (65%), iso-thujone (8%), fenchone (8%), and sabinene (5%) [23]. In contrast, essential oils obtained from dried leaves showed higher yields, ranging from 1.4% to 4%, with thujone as the principal component, consisting of α-thujone (85%) and β-thujone (15%) [24]. By applying an appropriate drying method, it might be possible to achieve higher essential oil yields while preserving valuable components in *T. occidentalis* leaf oils. Dănilă et al. [25] also mentioned that air drying at room temperature might be a better method for obtaining a high oil yield and bioactive components of α-thujone and β-thujone for *T. occidentalis*. The effects of drying on essential oils have been studied in aromatic plants such as basil [26], spearmint [27], and oregano [28] to identify suitable pre-treatment conditions for the commercial production of essential oils. Further research is needed to increase the oil yield and bioactive components of *T. occidentalis* leaf oils in relation to post-harvest drying conditions.

As shown in Table 1, *T. occidentalis* oil contained 49 components representing 96.84% of the total oils, predominantly monoterpenes and oxygenated monoterpenes. Specifically, the major constituents were α-thujone (45.52%), fenchone (10.17%), and β-thujone (6.23%). The components identified by GC–MS in *T. occidentalis* essential oils were consistent with previous reports, with α-pinene, sabinene, α-thujone, β-thujone, and fenchone identified as the major constituents [1]. To date, there are no reported data on *T. occidentalis* leaf oils originating from South Korea. Since essential oils composition and yield varies depending on geographical origin and edaphoclimatic conditions [2,29]. For example, Svajdlenka, Má, rtonfi, Tomasko, Grancai and Nagy [29] reported that the main compounds in *T. occidentalis* oil from Slovakia were α-thujone, β-thujone, and fenchone, accounting for more than 50% of the total oils. In contrast, leaf essential oils from Tunisia were dominated by α-pinene (34.4%), and cedrol (13.17%), together comprising 47.57% of the total oils [30]. Further research is needed to screen *T. occidentalis* populations from diverse sites in South Korea to identify superior cultivars for commercial production.

### 3.2. Effects of the Length of the DT on the Oil Yield and Chemical Profile of T. occidentalis Oils

For successful commercialisation, distillation parameters must be clearly established in advance to ensure that the essential oil meets market requirements. Although DT is a simple and well-established process, optimal conditions (e.g., time and steam rate) must be determined for each plant material, as they cannot be generalised [31]. Moreover, terpenoids in essential oils are prone to thermolability and may be easily oxidised or hydrolysed during distillation and handling [32]. As shown in Figure 2, the leaf oil yield of *T. occidentalis* peaked at 40 and 80 min (0.54 ± 0.04% and 0.59 ± 0.02%, respectively) and then declined with prolonged DT, dropping 0.05 ± 0.01% at 480 min. Despite the continued increase in total oil volume at longer DTs, the incremental gain after 120 min was relatively small, indicating diminishing returns in extraction efficiency. Compared with *T. occidentalis* leaf oils extracted until no further oil could be obtained (26 h; oil yield: 2.20% DW), the fractional oil yield obtained at 80 min (0.59%) accounted for only 26.8% of the total extractable oil from the raw materials. Kaspute et al. [33] emphasised that the choice of extraction method for essential oils is critical for preserving key bioactive compounds and promoting sustainability by valorising plant biomass waste. For example, microwave-assisted extraction achieves higher yields compared to traditional methods, with some reports indicating increases of up to 40% in essential oil yield [34]. Therefore, applying advanced extraction technologies to *T. occidentalis* oils may facilitate the development of a sustainable, economically viable industry for commercial production by reducing DT.

As shown in Table 2, monoterpenes exhibited a decreasing trend with increasing DTs, whereas higher-molecular-weight compounds, such as sesquiterpenes and diterpenes, showed a gradual increase. Zheljazkov et al. [35] reported a similar trend. Specifically, the concentrations of linalool (0.05–0.237%) and methyl chavicol (0.70–1.38%) were highest at 5 min (the shortest DT) and progressively decreased with prolonged distillation. These compounds, which have relatively lower boiling points, are preferentially eluted during the early stage of distillation, resulting in higher initial concentrations. In contrast, the yield of trans-anethole at 360 and 480 min was higher than that at shorter DTs. Hence, observed compositional shift according to DTs can be explained by differences in volatility, boiling point, and molecular weight among terpene classes [36]. Briefly, highly volatile compounds are rapidly released and co-distilled with steam, whereas compounds with lower volatility and higher molecular weights are recovered more gradually over extended distillation periods. Simard et al. [37] investigated the effect of steam DT on *T. occidentalis* oils collected at 0, 30, 60, 90, 120, 180, and 300 min. During distillation, α-thujone increased slightly during the early stage (0–30 min) and subsequently decreased from 55.55% to 42.22% at 300 min, whereas β-thujone exhibited only minor variation (6.77–8.44%). Consequently, the total thujone content (α- + β-thujone) declined from a maximum of 63.92% at 30 min to 49.34% at 300 min, indicating that prolonged distillation reduced the relative abundance of thujones. A similar trend was observed in the present study (Table 2), although a different distillation method was employed. Specifically, the total thujone content decreased from 71.88% at 1 min to 1.08% at 480 min as DT increased. These findings suggest that thujones are preferentially recovered during the early stages of hydrodistillation and become progressively depleted in later fractions due to their higher volatility.

DT can be manipulated to selectively enhance specific bioactive compounds in the essential oil. Lavender essential oil provides a representative example of this approach. For instance, a higher concentration of camphor (9.06–9.22%) can be obtained when dried lavender flowers are distilled for 7.5–15 min. In contrast, essential oil with a higher content of linalyl acetate (35.4–37.3%) is achieved when the plant material is distilled for at least 30 min [38]. Furthermore, Palmieri et al. [39] emphasised that DT can be adjusted according to the intended application of the essential oil, as it influences not only the chemical composition by selectively extracting different components and thereby increasing variability but also its biological activity.

DT can be adjusted to target specific components in *T. occidentalis* oils. For example, to obtain higher levels of caryophyllene (3.10 ± 0.07%), a DT of at least 360 min is required. Caryophyllene is known for its notable anticancer and analgesic properties [40], and thus, oils enriched in this compound may be considered of higher functional value. In contrast, thujone, a major monoterpene constituent of the oil, is widely used pharmacologically as an active ingredient in nasal decongestants and cough suppressants, as well as in perfumes, shoe polishes, and soaps. To maximise thujone content in *T. occidentalis* oils, leaf oils should be distilled for less than 120 min, during which approximately 50% of the total oil yield is recovered. Further research is required to evaluate the economic implications of DT, including labour, energy consumption, and production costs for commercial-scale production. For industrial applications, an economic analysis of the process is a significant factor in the final decision-making process [41].

### 3.3. Multivariate Analysis of DT-Dependent Chemical Composition and Fragrance Characteristics of T. occidentalis Oils

A multivariate analysis revealed clear differences in the fragrance characteristics of *T. occidentalis* oils across distillation stages (early, mid, and late), driven by distinct chemical compositions (Figure 3 and Figure 4). In cosmetic applications, fragrance plays a key role in enhancing the attractiveness of cosmetic products, as mesmerising scents influence user comfort, perceived product efficacy, and the overall evaluation of the cosmetics [42]. The PCA score plot showed well-separated clusters corresponding to each stage, with PC1 (68.4%) primarily distinguishing early-stage oils from late-stage oils, while mid-stage oils occupied an intermediate position (Figure 3). This separation was further supported by HCA analysis, which grouped the samples into three distinct compositional profiles.

Monoterpenes and ester compounds, which are known to contribute to fresh and highly volatile top-note aromas, are typically extracted at the early stages of distillation, whereas sesquiterpenes and oxygenated sesquiterpenes impart woody, balsamic, and long-lasting aroma characteristics due to their lower volatility [43,44]. As shown in Figure 4, early-stage oils were characterised by high levels of monoterpenes, particularly α-terpineol. These compounds are typically associated with fresh, sharp, and camphoraceous fragrance notes [45,46]. Yet, late-stage oils were dominated by sesquiterpenes and diterpenes, including caryophyllene, α-cadinol, α-eudesmol, hibaene, and rimuene, as confirmed by both PCA loading vectors and heatmap clustering (Figure 3 and Figure 4). These higher-molecular-weight compounds are typically less volatile and contribute to deep, woody, balsamic, and long-lasting base notes [47,48,49]. The enrichment of these compounds in the late stage might indicate a shift toward heavier and more persistent fragrance characteristics.

Overall, the results demonstrate a clear transition in fragrance profile from fresh and camphoraceous (early) to balanced and herbal (mid) and finally to woody and balsamic (late), corresponding to the extended DT of compounds with increasing molecular weight and decreasing volatility. This stage-dependent variation underscores the importance of DT in tailoring both the chemical composition and sensory properties of *T. occidentalis* oils. Additionally, the results of this laboratory-scale study cannot be directly extrapolated to industrial essential oil production, as industrial distillation systems operate under substantially different process conditions and equipment scales, which may influence extraction efficiency and chemical composition [50].

### 3.4. Safety Assessment of T. occidentalis Oils Under IFRA Regulatory Guideline

Compared with food and pharmaceutical products, the regulatory framework governing essential oils in cosmetic applications is relatively less strict. Consequently, the cosmetics industry bears significant responsibility for ensuring product safety, maintaining high quality standards, and providing transparent information regarding the use of essential oils [42]. To minimise the risk of adverse effects associated with the use of essential oils in cosmetics, the IFRA defined which essential oils and which of their constituents represent a potential allergy risk. IFRA also established the maximum concentration of essential oils in order to produce safe cosmetic products [51].

Essential oils may be safe at low concentrations, but become toxicity to humans at high concentrations represented as lethal dosages [52]. Few of the well-known essential oils and their bioactive components have toxic effects on human at high concentrations. For example, exposure to essential oils such as wormwood oil (*Artemisia absinthium*), *Mentha pulegium*, calamus oil (*Acorus calamus*), and mustard oil (*Brassica nigra*) containing thujone, pulegone, β-asarone and allyl isocyanate, respectively have toxic effect in humans [53]. Therefore, essential oils should be used with great care, taking into account appropriate precautions regarding the concentration used, the method of application (route of administration), the target consumer, the major components of the oils, and their toxicological profiles [16].

Thujone is a monoterpene found in many plants, including *T. occidentalis*. *Thuja*’s use as a medicinal herb is its content of thujone, which is reported to be the toxic agent of many fresh plants, especially intake for the human diet [54]. For example, the use of thujone is regulated by the European Parliament and Council and the European Medicines Agency [55]. As a fragrance, thujone is a regulated compound, and the estimated concentration is 55%, according to the IFRA 49th amendment [56]. The isomers α-thujone and β-thujone are monoterpene ketones, with the following IUPAC name: (1S,4R,5R)-4-methyl-1-(propane-2-yl) bicyclo (3.1.0) hexan-3-one [55]. As shown in Table 4, the maximum allowable use levels of *T. occidentalis* essential oils in finished products were estimated across IFRA Categories 1–12 based on the thujone content of oils obtained at 14 DTs. These results clearly demonstrate that DT is a critical parameter influencing not only the chemical composition but also the regulatory and formulation feasibility of *T. occidentalis* oils. For safe industrial application, medicinal crops should be validated through extensive pharmacological studies, demonstrating a wide range of biological activities and providing evidence for their efficacy through both in vitro and in vivo studies [57]. Hence, extended DT effectively reduces thujone-related restrictions, thereby broadening the potential application range of the oils in cosmetic and fragrance products.

## 4. Materials and Methods

### 4.1. Chemical Reagents

C7–C40 saturated alkanes standard mix (Lot #LRAC3115) was sourced from Sigma-Aldrich, St Louis, MO, USA. Anhydrous sodium sulfate (98.5%, Samchun, Seoul, Republic of Korea) was used.

### 4.2. Plant Material and Essential Oil Hydrodistillation

*T. occidentalis* samples were collected from Hoseo University, Asan campus, Chuncheongam-do (36.8286° N, 127.1805° E), Republic of Korea (30 October 2025). The characteristics of sampling site were obtained from weather extrapolation. The database and tool are a comprehensive archive of climate data recorded by the Korea Meteorological Administration National Climate Data Centre. Based on climate data for October 2025, the mean minimum, mean, and maximum temperatures were 12.1 °C, 15.9 °C, and 27.5 °C, respectively, with a total precipitation of 171.6 mm [58].

*T. occidentalis* trees (approximately 8–12 years old; plant height 3–4 m) were used in this study. The trial was conducted using a randomised complete block design (RCBD) with three blocks. Especially, plant materials were collected as pooled samples from multiple trees growing under similar environmental conditions to minimise individual tree variation. Approximately 2 kg of *T. occidentalis* samples were collected from individual trees within each block. Plant materials were obtained through pruning and thinning processes as a non-destructive method. Only healthy needles, free from drying, discolouration, and pest or disease damage, were carefully selected and collected for the experiment. The sampled plant was cut and labelled in the field, then stored on ice in a thermal-resistant container for transport to the laboratory. The essential oil was extracted from the needles, along with small twigs (diameter < 1 cm), of *T. occidentalis*. The needles were separated from larger branches before extraction, and only needles and fine twigs were included as the extraction material.

The samples were then weighed, and sub-samples were dried at 105 °C for 24 h to determine oven-dry weights (ODW). The remaining materials were packed in plastic bags and stored at −18 °C until hydrodistillation for extraction.

The essential oils were extracted from the leaves via hydrodistillation using Clevenger apparatus. Essential oils extraction was performed at the National Institute of Forest Science (Seoul, Republic of Korea). Specifically, each sample (1.6 kg) was mixed with distilled water (DW) in a ratio of 1:7 (kg:L). Samples soaked in the DW were heated using a heating mantle (Model: MS-DM608, Serial number: 201602, Misung Scientific Co., Ltd., Yangju, Republic of Korea) to approximately 102 °C, which corresponds to the boiling temperature of the water-plant mixture under the experimental conditions. Distillation was continued until no further oil was obtained (26 h), and the resulting sample was used as the control.

The current experimental design was modified according to previous studies [59,60]. To investigate the DT effect on essential oils, samples were taken at 1, 3, 5, 10, 20, 40, 80, 120, 160, 200, 240, 280, 360, and 480 min. Specifically, essential oil fractions were collected at 14 sequential DT intervals (0–1 min, 1–3 min, 3–5 min, 5–10 min, 10–20 min, 20–40 min, 40–80 min, 80–120 min, 120–160 min, 160–200 min, 200–240 min, 240–280 min, 280–360 min, and 360–480 min), along with a control. The internal volume of the collection arm is minimal, and no visible oil residue remained after collection. The oils were obtained and analysed separately at the 14 different DTs. Each fraction was taken from the same Clevenger apparatus. The yield of essential oils was calculated using the following equation.

Essential oil yield (%) = [essential oils distilled (mL)/sample weight (ODWg)] × 100%

The essential oils were dehydrated using an anhydrous sodium sulfate (98.5%, Samchun, Seoul, Republic of Korea) and stored in a refrigerator at 4 °C until use.

### 4.3. Analysis of Gas Chromatography–Mass Spectrometry (GC-MS)

The volatile constituents of the essential oils were analysed using GC–MS (7890B GC system coupled with a 5977A MSD, Agilent Technologies, Inc., Santa Clara, CA, USA) equipped with a VF-5MS capillary column (60 m × 0.25 mm, 0.25 µm; Agilent Technologies). The temperature of the GC injector was set to 280 °C, and the flow rate of the helium carrier gas was 2.0 mL/min. The initial oven temperature was 50 °C (5 min), followed by a temperature increase to 65 °C (30 min) at 10 °C/min. Thereafter, the temperature was raised to 210 °C (10 min) at 5 °C/min and, finally, to 305 °C (5 min) at 20 °C/min. The MS was conducted in the electron ionisation mode (70 eV). The ion source and interface temperature were set to 270 °C and 250 °C, respectively, and a mass range of 35–550 amu was recorded in the full scan mode. The Kovats retention index (KI) of the individual compounds was evaluated by comparing their relative retention times with those of an n-alkanes mixture (C8–C30, Sigma-Aldrich, St. Louis, MO, USA) in a VF-5MS column. The volatile constituents were identified by comparing their calculated KIs with the reported values (e.g., NIST Chemistry WebBook). All GC–MS analyses were conducted at the National Instrumentation Centre for Environmental Management (NICEM), Seoul National University, Seoul, Republic of Korea.

### 4.4. Multivariate Statistical Analysis

The multivariate statistical analysis were performed according to references [61,62] with modification. Multivariate statistical analysis was conducted to evaluate the chemical composition of *T. occidentalis* oils. For PCA visualisation, samples were grouped into early (1–10 min), mid (20–80 min), and late (120–480 min) stages. This grouping was further supported by PCA and HCA results, which showed clear separation of samples into three distinct compositional clusters (Figure 3 and Figure 4).

GC–MS data, expressed as relative peak area percentages, were organised into a data matrix and normalised to total ion abundance, followed by autoscaling (mean-centered and unit variance). PCA was performed as an unsupervised method to visualise compositional differences among essential oil samples extracted at different DTs. PCA score plots were used to assess sample clustering, while PCA biplots illustrated the contribution of individual volatile compounds to sample separation. The variance explained by each principal component was calculated. HCA was carried out using auto-scaled data, with Euclidean distance and Ward’s linkage method [63,64]. Heatmaps were generated to visualise relative compound abundance across samples, with rows representing volatile compounds and columns representing biological replicates. Analyses were performed using MetaboAnalyst software (version 6.0; University of Alberta, Edmonton, AB, Canada). Additionally, the odour descriptions of the identified compounds were assigned based on previously published literature [43,44,48,49].

### 4.5. Safety Assessment of T. occidentalis Oil, Based on IFRA Standards

The maximum allowable use level of essential oils in finished products across IFRA Categories 1–12 was estimated by dividing the IFRA maximum acceptable concentration of thujone by the thujone content (%) of each fractional oil sample. The maximum allowable essential oil level was calculated using the following equation:

Maximum essential oil level (%) = IFRA category limit (%)/Thujone content in oil (%) × 100

The IFRA category limits were obtained from Table 5 [56]. Values exceeding 100% were capped and expressed as >100, indicating no practical restriction. Values below 0.01% are expressed as <0.01. Calculations were performed using mean thujone values obtained from triplicate analyses.

### 4.6. Statistical Analysis

Data is presented as means ± SD (*n* = 3). One limitation of this study is that the sample obtained by exhaustive hydrodistillation (26 h) was analysed as a single replicate (*n* = 1), due to the limited availability of plant material. Consequently, the control was not included in statistical analyses and was used only as a qualitative reference.

The difference in oil yield and chemical profile of oils was analysed using one-way ANOVA followed by post hoc Tukey’s multiple range tests (Version 9.4; SAS Institute Inc., Cary, NC, USA). Furthermore, multivariate statistical analysis was performed using MetaboAnalyst software (version 6.0; University of Alberta, Edmonton, AB, Canada).

## 5. Conclusions

For commercial production, understanding DT is crucial for high efficiency within shortened processing times, along with industrial applicability and bioavailability. This study investigates the effects of 14 DTs (1–480 min) on the yield and chemical composition of leaf essential oils from *T. occidentalis*. In addition, a safety assessment was performed based on thujone content and regulatory limits for finished products under IFRA guidelines.

Oil yield of *T. occidentalis* at 40 and 80 min was significantly higher (0.54 ± 0.04% and 0.59 ± 0.02%, respectively) than those at other time points, indicating that most extractable volatile compounds were recovered within this interval; however, although total oil volume continued to increase at longer DTs, the incremental gain after 120 min was minimal, suggesting diminishing returns in extraction efficiency. PCA classified the samples into three stages: early (1–10 min), mid (20–80 min), and late (120–480 min), with clear compositional separation (PC1: 68.4%, PC2: 11.8%). Early stage samples were associated with oxygenated monoterpenes, particularly α-terpineol, whereas late-stage samples correlated with monoterpene hydrocarbons (limonene and α-fenchene) and higher molecular weight compounds such as kaur-16-ene. Mid-stage samples showed an intermediate chemical profile during distillation. The IFRA safety assessment showed that early-stage oils contained high thujone levels (67.19–71.88%), restricting their use, whereas longer DTs (≥200 min) reduced thujone content and increased allowable usage.

Overall, DT is a key parameter governing the yield, chemical composition, and sensory properties of *T. occidentalis* oil. To obtain higher levels of caryophyllene, a DT of at least 360 min is required, whereas maximising thujone content requires distillation for less than 120 min, during which approximately 50% of the total oil yield is recovered. Furthermore, DT directly influences the regulatory applicability and safe use of *T. occidentalis* oil. Future research is needed to investigate pilot-scale production, energy efficiency, extraction cost, and process optimisation for commercial applications.

## Figures and Tables

**Table 1:** The chemical profile of *T. occidentalis* oils.

	RT*	KI*	KI Ref*	Chemical Components	% in Oils
1	12.85	908	907	bornylene	0.09
2	13.86	927	931	α-thugene	0.73
3	14.29	935	939	α-pinene	1.71
4	14.81	945	953	α-fenchene	0.02
5	15.21	952	964	camphene	2.26
6	16.40	975	986	sabinene	2.83
7	16.73	981	981	β-pinene	0.15
8	17.21	990	991	myrcene	1.04
9	17.72	1000	1011	α-terpinene	0.01
10	18.32	1009	1013	*p*-cymene	0.12
11	18.95	1020	1027	limonene	0.90
12	19.44	1027	1031	1,8-cineole	0.94
13	19.71	1032	1039	β-phellandrene	1.83
14	21.50	1060	1060	γ-terpinene	1.38
15	23.25	1088	1085	terpinolene	0.37
16	23.60	1094	1087	fenchone	10.17
17	24.04	1101	1098	terpinolene	0.22
18	24.80	1113	1106	α-thujone	45.52
19	25.46	1123	1110	β-thujone	6.23
20	26.04	1131	1119	campholenal	0.07
21	27.02	1123	1121	p-menth-2-en-1-ol	0.17
22	27.48	1153	1151	camphor	2.40
23	28.00	1161	1157	camphene hydrate	0.22
24	28.13	1163	1165	endo-borneol	0.17
25	28.79	1173	1180	borneol	0.25
26	29.6	1185	1181	terpinene-4-ol	2.42
27	29.99	1191	1185	p-cymen-8-ol	0.25
28	30.55	1185	1189	α-terpineol	0.45
29	31.94	1220	1217	fenchyl acetate	0.21
30	33.30	1240	1260	chavicol	0.22
31	34.72	1261	1266	neothujyl acetate	0.72
32	35.29	1270	1270	cyclofenchone	0.20
33	36.44	1287	1291	bornyl acetate	2.20
34	41.24	1356	1350	α-terpinyl acetate	1.16
35	43.18	1384	1381	cedrene	0.12
36	45.55	1429	1432	caryophyllene	2.27
37	47.45	1462	1460	cis-thujopsene	0.12
38	47.61	1476	1466	humulene	0.17
39	48.55	1498	1495	α-muurolene	0.13
40	49.49	1526	1524	δ-cadinene	0.09
41	51.74	1595	1597	caryophyllene oxide	1.05
42	52.34	1616	1605	β-oplopenone	0.10
43	52.58	1625	1648	acorenol	0.28
44	53.81	1670	1666	α-cadinol	0.15
45	54.22	1685	1674	α-eudesmol	0.16
46	59.68	1926	1927	rimuene	1.38
47	60.38	1961	1951	beyerene	0.06
48	60.58	1971	1962	hibaene	2.57
49	63.24	2111	2082	kaur-16-ene	0.56
	Total identified components (%)				96.84
	Unknown components (%)				3.16

RT*: Retention time (min), KI*: Kovats index, KI ref*: KI reference from NIST Chemistry WebBook.

**Table 2:** The effect of DT on the selected components in *T. occidentalis* oils.

DT (min)	Monoterpenes				Sesquiterpenes	Diterpenes	
	Sabinene	Fenchone	α-Thujone	β-Thujone	Caryophyllene	Hibaene	Rimuene
1	4.47 ± 0.31 ^a^	15.26 ± 0.01 ^a^	60.30 ± 1.87 ^ab^	6.89 ± 0.85 ^a^	2.01 ± 0.55 ^b^	0.27 ± 0.06 ^f^	0.17 ± 0.04 ^i^
3	4.17 ± 0.39 ^a^	15.55 ± 0.04 ^a^	64.3 ± 1.32 ^a^	7.58 ± 0.54 ^a^	2.14 ± 0.05 ^b^	0.62 ± 0.11 ^ef^	0.43 ± 0.09 ^gh^
5	2.88 ± 0.09 ^b^	15.88 ± 0.59 ^a^	61.26 ± 0.71 ^ab^	6.78 ± 0.34 ^a^	2.05 ± 0.01 ^b^	0.54 ± 0.01 ^ef^	0.44 ± 0.06 ^gh^
10	2.83 ± 0.12 ^b^	15.98 ± 0.85 ^a^	60.93 ± 1.84 ^ab^	7.55 ± 0.41 ^a^	2.06 ± 0.01 ^b^	0.70 ± 0.70 ^ef^	0.45 ± 0.04 ^g^
20	2.99 ± 0.74 ^b^	15.14 ± 0.29 ^a^	62.22 ± 0.15 ^a^	7.25 ± 0.21 ^a^	1.90 ± 0.11 ^b^	0.54 ± 0.08 ^ef^	0.33 ± 0.02 ^h^
40	1.91 ± 0.14 ^c^	13.54 ± 0.18 ^b^	60.51 ± 0.83 ^ab^	7.54 ± 0.04 ^a^	1.90 ± 0.01 ^b^	0.61 ± 0.03 ^ef^	0.35 ± 0.02 ^gh^
80	1.35 ± 0.23 ^cd^	10.61 ± 0.23 ^c^	56.64 ± 0.40 ^b^	7.60 ± 0.07 ^a^	2.08 ± 0.10 ^b^	0.74 ± 0.02 ^ef^	0.42 ± 0.02 ^h^
120	1.25 ± 0.06 ^cd^	6.68 ± 0.71 ^d^	44.9 ± 2.61 ^c^	6.76 ± 0.36 ^a^	2.10 ± 0.04 ^b^	1.21 ± 0.02 ^de^	0.78 ± 0.02 ^f^
160	0.99 ± 0.01 ^d^	3.50 ± 0.64 ^e^	28.16 ± 2.97 ^d^	4.70 ± 0.46 ^b^	2.10 ± 0.08 ^b^	1.99 ± 0.01 ^d^	1.02 ± 0.04 ^e^
200	0.98 ± 0.04 ^d^	1.74 ± 0.44 ^f^	14.81 ± 3.14 ^e^	2.67 ± 0.57 ^c^	1.91 ± 0.20 ^b^	2.89 ± 0.58 ^c^	1.32 ± 0.02 ^d^
240	1.22 ± 0.03 ^cd^	0.84 ± 0.15 ^fg^	6.69 ± 0.99 ^f^	1.28 ± 0.31 ^d^	2.09 ± 0.11 ^b^	3.11 ± 0.21 ^c^	1.58 ± 0.03 ^c^
280	1.59 ± 0.08 ^cd^	0.50 ± 0.07 ^g^	2.99 ± 0.28 ^fg^	0.57 ± 0.00 ^d^	2.38 ± 0.41 ^b^	4.68 ± 0.08 ^b^	1.68 ± 0.02 ^c^
360	1.07 ± 0.03 ^d^	0.42 ± 0.06 ^g^	1.65 ± 0.08 ^g^	0.33 ± 0.08 ^d^	3.10 ± 0.07 ^a^	4.90 ± 0.20 ^b^	1.87 ± 0.02 ^b^
480	0.95 ± 0.32 ^d^	0.39 ± 0.05 ^g^	0.90 ± 0.04 ^g^	0.18 ± 0.02 ^d^	3.12 ± 0.10 ^a^	6.39 ± 0.78 ^a^	2.01 ± 0.02 ^a^

Values were expressed as means ± SD (*n* = 3). Values with different superscript letters were significantly different at *p* < 0.05 as analysed by Tukey’s multiple range test of specialised fractional distillation for the selected components in oils.

**Table 3:** Major contributors to PCA clustering based on loading values of PC1 and PC2.

Rank	Major Contributors to PC1	Loading Value	Major Contributors to PC2	Loading Value
1	α-Terpinene	0.1816	endo-Borneol	−0.3615
2	α-Terpineol	0.1532	α-Fenchene	−0.2288
3	Fenchone	0.1265	3-Carene	−0.212
4	α-Thujone	0.1149	α-Terpineol	−0.2016
5	β-Thujone	0.1012	Camphor	−0.1273
6	Camphor	0.0829	β-Thujone	−0.1228
7	β-Pinene	−0.0526	α-Thujone	−0.1208
8	Sabinene	−0.0545	α-Pinene	0.1157
9	Linalool	−0.0761	Linalool	0.1341
10	endo-Borneol	−0.0872	α-Thugene	0.1394

Only the major contributors with the highest absolute loading values are presented.

**Table 4:** Maximum allowable use level (%) of *T. occidentalis* oils across IFRA categories based on thujone content.

DT (min)	Thujone in Oil (%)	Cat. 1	Cat. 2	Cat. 3	Cat. 4	Cat. 5(A)	Cat. 5(B)	Cat. 5(C)	Cat. 5(D)	Cat. 6	Cat. 7(A)	Cat. 7(B)	Cat. 8	Cat. 9	Cat. 10(A)	Cat. 10(B)	Cat. 11(A)	Cat. 11(B)	Cat. 12
1	71.88	0.15	>100	0.02	>100	<0.01	>100	<0.01	>100	0.01	>100	0.01	37.37	0.35	37.37	0.59	0.9	0.59	>100
3	69.47	0.16	>100	0.02	>100	<0.01	>100	<0.01	>100	0.01	>100	0.01	36.12	0.36	36.12	0.61	0.87	0.61	>100
5	68.48	0.16	>100	0.02	>100	<0.01	>100	<0.01	>100	0.01	>100	0.01	35.6	0.37	35.6	0.62	0.86	0.62	>100
10	68.05	0.16	>100	0.02	>100	<0.01	>100	<0.01	>100	0.01	>100	0.01	35.38	0.37	35.38	0.62	0.85	0.62	>100
20	68.04	0.16	>100	0.02	>100	<0.01	>100	<0.01	>100	0.01	>100	0.01	35.38	0.37	35.38	0.62	0.85	0.62	>100
40	67.19	0.16	>100	0.02	>100	<0.01	>100	<0.01	>100	0.02	>100	0.02	34.93	0.37	34.93	0.63	0.84	0.63	>100
80	64.24	0.17	>100	0.03	>100	<0.01	>100	<0.01	>100	0.02	>100	0.02	33.4	0.39	33.4	0.66	0.8	0.66	>100
120	51.66	0.21	98.62	0.03	>100	<0.01	>100	<0.01	>100	0.02	>100	0.02	26.86	0.48	26.86	0.82	0.65	0.82	>100
160	32.86	0.33	62.73	0.05	>100	<0.01	>100	<0.01	>100	0.03	>100	0.03	17.08	0.76	17.08	1.29	0.41	1.29	>100
200	17.48	0.63	33.37	0.1	>100	0.01	>100	<0.01	>100	0.06	>100	0.06	9.09	1.43	9.09	2.42	0.22	2.42	>100
240	7.97	1.38	15.22	0.21	>100	0.01	>100	0.01	>100	0.13	>100	0.13	4.14	3.14	4.14	5.31	0.1	5.31	>100
280	3.56	3.09	6.8	0.47	>100	0.03	>100	0.02	>100	0.29	>100	0.29	1.85	7.02	1.85	11.89	0.04	11.89	79.93
360	1.98	5.56	3.78	0.85	>100	0.06	>100	0.03	>100	0.51	>100	0.51	1.03	12.63	1.03	21.37	0.02	21.37	44.45
480	1.08	10.19	2.06	1.55	90.2	0.11	>100	0.05	>100	0.94	>100	0.94	0.56	23.15	0.56	39.18	0.01	39.18	24.25

Cat.: Category, Calculations were performed using mean values of thujone content. Values represent the maximum allowable concentration (%) of *T. occidentalis* essential oil in finished products, calculated based on IFRA limits for thujone and the mean thujone content of each fractional oils. Values exceeding 100% are expressed as >100, indicating no practical restriction. Values below 0.01% are expressed as <0.01.

**Table 5:** Guidance for the use of thujone across IFRA standard according to product type (Category 1–12).

Category		Product Type	%	Category		Product Type	%
Cat. 1		Products applied to the lips	0.11	Cat. 7	(A)	Rinse-off products applied to the hair with some hand Contact	0.24
Cat. 2		Products applied to the axillae	0.21	Cat. 7	(B)	Leave-on products applied to the hair with some hand contact	0.24
Cat. 3		Products applied to the face/body using fingertips	0.032	Cat. 8		Products with significant anogenital exposure	0.0053
Cat. 4		Products related to fine fragrance	1.4	Cat. 9		Products with body and hand exposure, primarily rinse off	0.13
Cat. 5	(A)	Body lotion products applied to the body using the hands (palms), primarily leave on	0.095	Cat. 10	(A)	Household care products with mostly hand contact	0.13
Cat. 5	(B)	Face moisturiser products applied to the face using the hands (palms), primarily leave on	0.032	Cat. 10	(B)	Household care products with mostly hand contact, including aerosol/spray products (with potential leave-on skin contact)	0.22
Cat. 5	(C)	Hand cream products applied to the hands using the hands (palms), primarily leave on	0.016	Cat. 11	(A)	Products with intended skin contact but minimal transfer of fragrance to skin from inert substrate without UV exposure	0.0053
Cat. 5	(D)	Baby creams, baby oils and baby talc	0.0053	Cat. 11	(B)	Products with intended skin contact but minimal transfer of fragrance to skin from inert substrate with potential UV exposure	0.0053
Cat. 6		Products with oral and lip exposure	0.095	Cat. 12		Products not intended for direct skin contact, minimal or insignificant transfer to skin	9.5

## Data Availability

The original contributions presented in this study are included in the article. Further inquiries can be directed to the corresponding author.

## Abbreviation

The following abbreviation is used in this manuscript:

DT	Distillation Time
